# P01.41. Melittin inhibits VEGF-A-induced tumor growth and angiogenesis through blocking VEGFR-2 and COX-2 in allograft tumor model and endothelial cells

**DOI:** 10.1186/1472-6882-12-S1-P41

**Published:** 2012-06-12

**Authors:** J Huh, J Kang, Y Baek, D Choi, D Park, J Lee

**Affiliations:** 1 Kyung Hee University, Seoul, Republic of Korea

## Purpose

To evaluate the *in vivo* as well as *in vitro* anti-angiogenesis effects of melittin, a major polypeptide in bee venom, and to elucidate its molecular mechanisms with a special focus on VEGFR-2 mediated COX-2 and MAPK pathways in VEGF-A-induced Lewis lung cancer (VEGF-A-hm LLC) model and human lymphatic endothelial cells (VEGF-A-HLECs).

## Methods

We investigated the functional specificity of melittin as an angiogenesis inhibitor using VEGF-A-induced *in vitro* models and an *in vivo* lung metastasis mouse model.

## Results

Injection of 0.5mg/kg and 5mg/kg of melittin suppressed tumor growth by 25.30% and 56.92%, respectively; these results are superior to those obtained for mice treated with the COX-2 inhibitor, NS398. Melittin reduced tumor cell proliferation (PCNA), microvessel density (MVD), expression of cyclooxygenase-2 (COX-2), VEGF-A, and VEGFR-2, but did not affect VEGFR-1, in VEGF-A-induced hm LLC tumors. Similarly, the COX-2 inhibitor NS398 significantly inhibited proliferation, MVD, COX-2, VEGF-A, and VEGFR-2 expression in the tumor section, supporting the role of COX-2 in melittin-induced inhibition of angiogenesis. Melittin significantly inhibited VEGF-A-induced proliferation and tube formation in the endothelial cells. Melittin inhibited phosphorylation of ERK 1/2, JNK and p38 MAPK in a dose-dependent manner in VEGF-A-HLECs. p38 inhibitor SB203580 abolished the down regulation of COX-2 and VEGF-A and anti-proliferative activity induced by melittin.

## Conclusion

These results suggest that melittin suppresses VEGF-A-induced tumor growth and angiogenesis via VEGFR-2 mediated COX-2 and the MAPK-dependent pathway.

